# Effects of Sensorimotor Training on Transversus Abdominis Activation in Chronic Low Back Pain Patients

**DOI:** 10.3390/jpm13050817

**Published:** 2023-05-11

**Authors:** Felix Marchand, Kevin Laudner, Karl-Stefan Delank, René Schwesig, Anke Steinmetz

**Affiliations:** 1 Department of Orthopedic and Trauma Surgery, Martin-Luther-University Halle-Wittenberg, 06120 Halle, Germany; f.marchand@jhwaf.de (F.M.); stefan.delank@uk-halle.de (K.-S.D.); rene.schwesig@uk-halle.de (R.S.); 2 Department of Orthopedic and Trauma Surgery, Josephs-Hospital Warendorf, 48231 Warendorf, Germany; 3 Department of Health Sciences, Hybl Sports Medicine and Performance Center, University of Colorado, Colorado Springs, CO 80918, USA; klaudner@uccs.edu; 4 Department of Trauma, Reconstructive Surgery and Rehabilitation Medicine, University Medicine Greifswald, Physical and Rehabilitation Medicine, 17475 Greifswald, Germany

**Keywords:** Galileo^®^, Posturomed^®^, chronic low back pain, sensorimotor training, transversus abdominis activation, physiotherapy, rehabilitation

## Abstract

(1) Background: The aim of this study was to investigate and compare the effect of sensorimotor training on transversus abdominis activation. (2) Methods: Seventy-five patients with chronic low back pain were randomly assigned to one of three groups (whole body vibration training using Galileo^®^, coordination training using Posturomed^®^, or physiotherapy (control)). Transversus abdominis activation was measured by using sonography pre- and post-intervention. Second, changes in clinical function tests and their correlation with the sonographic measurements were determined. (3) Results: All three groups showed an improvement in activation of the transversus abdominis post-intervention, with the Galileo^®^ demonstrating the largest improvement. There were no relevant (r > 0.5) correlations between activation of the transversus abdominis muscle and any clinical tests. (4) Conclusions: The present study provides evidence that sensorimotor training on the Galileo^®^ significantly improves the activation of the transversus abdominis muscle.

## 1. Introduction

Back pain is one of the largest health problems in Germany and the entire Western world [1,2]. Current studies clearly point to the high risk of chronic back pain, with 65% of first-time patients with non-specific back pain reporting a relapse within the first year [3]. About 10% of back pain patients report significant restrictions with work and quality of life [4]. If therapy is not successful after six weeks, a multidisciplinary assessment should be conducted to reduce the risk of chronicity. This includes the determination of specified therapy techniques such as multimodal pain therapy [5]. As such, sensory-motor training is usually conducted in addition to various physiotherapeutic, psychoeducational and psychotherapeutic measures.

The aim of sensorimotor training is to increase proprioceptive input and the subsequent motor response during dynamic situations. This can be achieved, for example, through exercises performed on unstable surfaces [6]. For patients with chronic non-specific back pain, the aim is to improve the intra- and inter-muscular coordination of the superficial and deep trunk muscles. In addition to the multifidi muscles, the pelvic floor and the diaphragm, and the transversus abdominis play an important role in the dynamic segmental stabilization of the lumbar spine [7,8,9]. The transversus abdominis exerts traction on the thoracolumbar fascia and thus, via co-contraction with the lumbar multifidi muscles, exerts a stabilizing effect on the spine [8,10]. Of all the abdominal muscles, the transversus abdominis has the highest activity in maintaining intra-abdominal pressure [11].

Sensorimotor training also increases the motor adaptability, segmental stability and overall performance of muscles, which thereby reduces pain frequency and intensity [12]. However, despite extensive research in recent decades, no concrete recommendations for the implementation of sensorimotor training have been derived regarding the exact form, frequency and intensity for optimal training effects [6,13].

The aim of this study was to investigate the effect of sensorimotor training on the Galileo^®^ and on the Posturomed^®^ have on transversus abdominis activation in patients with chronic low back pain during inpatient multimodal pain or complex therapy compared to conventional sensorimotor physiotherapy. Activation of the transversus abdominis was assessed using sonography and clinical tests.

## 2. Materials and Methods

### 2.1. Design

The study was conducted as a prospective, randomized and confirmatory intervention. Patients (n = 75) with chronic low back pain were randomly divided into two intervention groups (Galileo^®^ and Posturomed^®^) and a control group (conventional physiotherapy) with 25 patients per group. The participants were recruited from the patient collective of the conservative orthopedic department of the University Hospital Halle (Saale). All participants were patients with specialist indication for a two-week multimodal therapy for chronic low back pain (according to the National Treatment Guideline [5]). Participants had to be at least 18 years old and suffer from chronic low back pain that had been present for more than 12 weeks. Exclusion criteria included trauma, infection, tumor, spinal surgery history and pregnancy or breastfeeding. During the inpatient stay, the control group received six sessions of sensorimotor training using conventional physiotherapy, while the intervention groups received six units of sensorimotor training on the Galileo^®^ or the Posturomed^®^. Sonographic examination of the transversus abdominis and functional clinical tests were used to determine segmental stability pre- (T0) and post-intervention (T1).

The study was conducted according to the Declaration of Helsinki in the current version between July 2015 and January 2017. Ethical clearance was granted by the Institutional Ethics Committee (reference number: 2015-84), and written informed consent was obtained from all participants. Data regarding the effect of sensorimotor training on postural control and functional status have been previously published [14].

### 2.2. Sonography of Transversus Abdominis

To measure activity of the transversus abdominis, an ultrasound probe was positioned in transverse alignment on the patient’s right side, above the iliac crest, lateral to the anterior superior iliac spine and in the area of the mid-axillary line [15]. The sonographic images were then captured of the cross-section muscle thickness of the transversus abdominis. This measurement was taken under two conditions:

1. supine position with the abdominal muscles relaxed (50° hip flexion and 90° knee flexion) [16],
2. supine position during targeted contraction of the muscle using the “abdominal draw-in maneuver” (patient holds his/her breath after expiration and gently draws their navel inwards without moving the pelvis) [17].

By measuring muscle thickness in different starting positions, the absolute changes in thickness (cm) and the relative changes in muscle thickness (%) due to tension (abdominal draw-in maneuver) were able to be determined. The draw-in maneuver is considered a measure of the activation of the transversus abdominis. The percentage increase was then calculated using the following formula [16,18,19,20]:

$$\mathrm{Activation}\;=\frac{\left(\mathrm{muscle}\;\mathrm{thickness}\;\mathrm{relaxation}\;-\;\mathrm{muscle}\;\mathrm{thickness}\;\mathrm{tension}\right)}{\mathrm{muscle}\;\mathrm{thickness}\;\mathrm{relaxation}}$$

As in comparable studies, functional ultrasound of the transversus abdominis muscle was chosen due to its proven validity in the assessment of segmental stability in patients with chronic low back pain [17,18,21,22,23,24,25]. Sonography seems to have an advantage over surface EMG, which cannot adequately differentiate between the external abdominal oblique, internal abdominal oblique and transversus abdominal muscles [20,23,26].

### 2.3. Clinical Tests

Clinical tests were used to obtain a clinical picture of the quality of movement control as well as activity and coordination of the deep trunk muscles. Seven clinical tests (Table 1), with good to very good reliability (ICC range: = 0.687–0.895), were completed [24]. The tests were rated as “inconspicuous” or “conspicuous” in terms of performance.

### 2.4. Intervention

Sensorimotor training on the Galileo^®^ and the Posturomed^®^ are considered widely used therapies in daily clinical practice.

#### 2.4.1. Galileo^®^ Training

A Galileo^®^ Med M vibration platform (Novotec Medical GmbH, Pforzheim, Germany) was used for training the Galileo^®^ group (n = 25). This therapy platform uses a side-alternating movement pattern such as a seesaw with variable amplitude and frequency. For this training, a tilting movement of the pelvis generates a movement pattern, similar to a human gait, but much more frequent. The body reacts in a compensatory manner with rhythmic muscle contractions, which are reflex controlled from a frequency of approximately 10 Hz. This activates the muscles in the legs, abdomen and back throughout the torso. The frequency of the vibration plate is continuously adjustable between 5 and 30 Hz, as is the amplitude of ±4.5 mm. Low frequencies can be used for mobilization, medium for muscle function and coordination and high to increase muscle performance and endurance [14]. A total of six training sessions were carried out by the Galileo^®^ group under physiotherapeutic supervision by an experienced physiotherapist. The total training time per session was 15 min.

#### 2.4.2. Posturomed^®^ Training

The Bioswing Posturomed^®^ 202 platform (Haider Bioswing GmbH, Pullenreuth, Germany) was used for training the Posturomed^®^ group (n = 25). This is a sensorimotor therapy and training device with a 60 × 60 cm damped oscillating unstable surface. The surface, which is suspended from eight steel cables, enables progressively damped evasive movements. Different oscillation amplitudes (max. surface deflection mediolateral 25 mm (locked)/50 mm (unlocked); max. surface deflection anterior-posterior 25 mm (locked)/50 mm (unlocked)) can be achieved by unlocking additional ropes. Maximum surface deflection in the anterior–posterior directions (40 mm/80 mm) and oscillation frequencies (1.0 to 4.2 Hz) are enabled. This allowed for an ideal severity adjustment of the exercises [14]. A total of six training sessions were carried out by the Posturomed^®^ group under physiotherapeutic supervision by an experienced physiotherapist. The total training time per session was 15 min.

### 2.5. Statistics

The sample size and number of cases per intervention group were based on comparable studies [16,22]. According to Bortz [25], an effect size of 0.7 (α = 0.05; 1 − β = 0.8) requires 25 patients per intervention group. The significance level was set at *p* < 0.05 and η_p_^2^ ≥ 0.10. All data were analyzed using SPSS 28.0 for Windows (SPSS, Chicago, IL, USA).

Descriptively, means, standard deviations and 95% confidence intervals (95% CI) were calculated. A Chi-square test was used for comparison of gender distribution. Age and anthropometric data were compared using a univariate analysis of variance. For the evaluation of the clinical tests, the multivariate linear model was used, and effect sizes (d) were calculated according to Hartmann et al. [27]. For the evaluation of the sonographic thickness of the transversus abdominis, a post-hoc test was used in addition to the general linear model.

Correlations between the data of the clinical tests and the sonographic data were investigated by calculating the Pearson correlation coefficient.

## 3. Results

### 3.1. Participants

A total of 48 (64%) of the participants were female and 27 (36%) were male (Table 2). The Chi-square test showed an unequal gender distribution between the groups, but this was not statistically significant (*p* = 0.105). No significant differences between groups were found for age or the anthropometric parameters of height, body weight and body mass index (Table 2).

### 3.2. Sonography of Transversus Abdominis Muscle

The absolute muscle thicknesses (cm) of the transversus abdominis in the relaxed position changed minimally from T0 to T1 in all groups, while a slight increase in muscle thickness occurred from 0.30 ± 0.08 cm to 0.37 ± 0.11 cm in the Posturomed^®^ group (Table 3). Only muscle activation for a relevant group effect was calculated (*p* = 0.003; η_p_^2^ = 0.147). Relevant time and interaction effects were only detected for muscle contracted (*p* < 0.001; η_p_^2^ = 0.225) and muscle relaxed (*p* = 0.019; η_p_^2^ = 0.105).

The absolute muscle thicknesses (cm) of the transversus abdominis in the tensed position showed a slight increase from T0 to T1 in all three groups. However, there were no significant differences between the groups.

**Figure 1:**
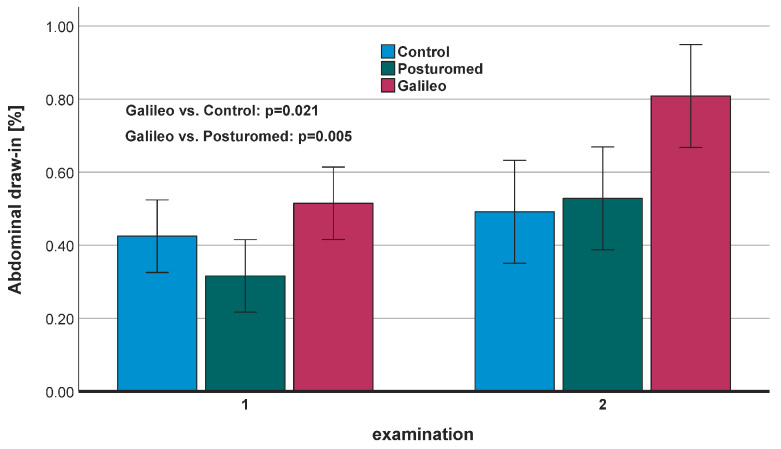
Targeted activation of the transversus abdominis using the abdominal draw-in maneuver. Increase in muscle thickness (%) at examination 1 and 2. The Galileo^®^ group showed a significant advantage compared with the Posturomed^®^ group and the control group.

At T0, the percentage increases in muscle thickness were 52% in the Galileo^®^ group, 32% in the Posturomed^®^ group and 43% in the control group. T1 showed muscle thickness increases of 81% in the Galileo^®^ group, 53% in the Posturomed^®^ group and 49% in the control group. After comparing the two measurement points, there was a clear improvement from 52% to 81% in the Galileo^®^ group and the Posturomed^®^ group (32% to 53%), while the control group improved from 43% to 49%. There were different time effects in all three groups, with the Galileo^®^ group having a significant group effect compared to the control and Posturomed^®^ groups (Figure 1).

### 3.3. Clinical Tests

Large effects were only seen during hip flexion in both the Posturomed^®^ group (d = 1.24) and the control group (d = 1.00; Table 4). Medium effects were found in the Galileo^®^ group during inspiration in the supine position, trunk flexion in the supine position, the Vele test and the Matthiaß test. The Posturomed^®^ group also showed medium effects for trunk flexion in the supine position and the single-leg stand. There were no medium effects in the control group.

No relevant correlations were found for activation of the transversus abdominis using sonography compared to any of the clinical tests (Table 5). The largest, but still not relevant relationship, was found during inspiration in a supine position (r = −0.276).

## 4. Discussion

The primary aim of this study was to investigate the effects of different forms of sensorimotor training on transversus abdominis activation in patients with chronic low back pain. When comparing the entire group of subjects (n = 75) with each of the three groups (n = 25), there was a homogeneous distribution of age and anthropometric data. Therefore, a good comparison of the data can be assumed. The unequal gender distribution in favor of females reflects the epidemiological situation in Germany where more women suffer from chronic low back pain than men [5]. Furthermore, the gender-specific differences in the different groups were not statistically significant.

Some authors have limited their sonographic examination to the absolute increase in thickness (hypertrophy) of the transversus abdominis achieved during 2–3 months of interventional muscle training [28,29]. These previous results showed that the increase in thickness was associated with a reduction in pain intensity and improvement in functional mobility. More recent work shows that, in addition to the absolute increase in thickness, the change in recruitment (i.e., activation) is crucial in chronic low back pain patient outcomes [9,16,19]. The intervention period in the present study was limited to two weeks and six therapy sessions, so no relevant muscle morphological changes were expected. A significant increase in the cross-sectional area of the entire muscle as well as individual muscle fibers, which results partially from the increase in the size and number of myofibrils, can only be expected after a training period of eight to twelve weeks [30].

The average muscle thickness of the transversus abdominis among all subjects during relaxation was approximately 0.34 cm. Comparably, Rankin et al. [31] described an average muscle cross-section of the transversus abdominis of 0.46 cm. However, this can differ significantly depending on gender, underlying diseases and age. The lower average thickness in the test group of the current study compared to the normal population is not surprising. All subjects included were patients with chronic low back pain. It has been well established that hypotrophy of the deep trunk muscles and especially of the transversus abdominis can be both a cause and a consequence of chronic low back pain [8,11,16,32,33]. In addition, the high proportion of female subjects and the average age of over 60 years within the subject population may possibly explain this deviation.

The mean values of the muscle thickness during tension showed a slight increase in all three groups T0 to T1. The positive time effects in the two intervention groups (Galileo^®^ and Posturomed^®^) were slightly higher than the control group but with no significant differences between groups. This indicates that both sensorimotor training on the Galileo^®^ and Posturomed^®^ and sensorimotor training through conventional physiotherapy can lead to improved contraction of the transversus abdominis. These results support previous research, which compared specialized trunk-stabilizing exercises with conventional physiotherapy in patients with chronic low back pain and found that muscle thickness of the transversus abdominis also served as a parameter for segmental trunk stability. Again, positive effects were seen in both groups with slight advantages among the intervention group, but with no significant group or interaction effects [26].

The results of this study indicate that even two weeks of sensorimotor training leads to an improvement in the targeted activation of the transversus abdominis in patients with chronic back pain. Sensorimotor training on the Galileo^®^ led to a significantly greater improvement in targeted muscle activation compared to sensorimotor training using the control and Posturomed^®^ group.

Cruz-Diaz et al. [34] used a similar study design to investigate the effect of Pilates training on transversus abdominis activation in patients with chronic low back pain and reported similar results. For this study, two different forms of Pilates training (Pilates on a mat and equipment-assisted Pilates) were compared with a control group. While no significant group and interaction effects were found for muscle thickness at rest, significant improvements were noted for the percentage increase in muscle thickness through targeted contraction (activation) in the intervention groups. In contrast to the present study, the intervention period was twelve weeks in total and the data were collected at three time periods. However, it is worth noting that the majority of the positive effects on the targeted activation of the transversus abdominis occurred between T0 and T1. This suggests that the improvement in activation of the transversus abdominis muscle is possible after a short intervention period and can occur independently of muscle morphological adaptations. Sensorimotor training primarily addresses the neuromuscular system, which controls inter- and intramuscular coordination. The improvement of muscle activation can be achieved through more effective recruitment of existing motor units and an increased frequency of nerve impulses. These neurological adaptations seem to have great potential already in the initial phases of training sessions [30]. This could explain the improved activation of the transversus abdominis despite the short intervention period.

Dong et al. [35] compared the muscle activity of different trunk muscles during the performance of motor exercises with and without the influence of whole-body vibration. These authors found that at a vibration frequency of 15 Hz, muscle activity increased by 190–247% of the multifidi muscles, erector spinae, external obliques, and rectus abdominis, but not on the transversus abdominis. However, a similar effect can also be assumed in the area of the deep abdominal muscles. This may partially explain the higher training effectiveness on the Galileo^®^.

Wang et al. [36] provided a review on the effect of whole-body vibration training in chronic back pain patients. Pain intensity and functional abilities were evaluated, but activity of the transversus abdominis was not included as a measure of lumbar segmental stability. Whole-body vibration therapies showed some significant improvements when compared to classical physiotherapy and conventional stability exercises, similar to the present study.

Another aim of the present study was to determine the effect of different forms of sensorimotor training on the performance of clinical tests in patients with chronic low back pain. All three groups showed a slight improvement in the performance of the clinical tests, which partially led to positive time effects, although significant interaction effects were not demonstrated. Since these seven clinical tests have not been previously validated as a measure for assessing lumbar segmental stability, the correlation coefficients between the functional clinical diagnostics and sonography were determined (r = −0.276–0.212). Unfortunately, no correlations were found in any of the clinical tests, so the interpretation of functional clinical diagnostics with regard to segmental stability in patients with chronic low back pain remains limited. Despite ongoing research, no reliable clinical tests for assessing segmental stability except the abdominal-draw-in maneuver while using a pressure pad have been established. When visually assessing movement quality and control during clinical examinations, there is already a problem of low inter- and intra-rater reliability, even among experienced healthcare professionals [37,38]. Therefore, more objective assessments of movement characteristics, such as sensor-based kinematic data, are needed. The potential here lies primarily in being able to assign underlying pathologies of the lumbar spine (e.g., segmental instability) to specific, abnormal movement patterns. This can help to identify the cause and effectiveness of treatments [39,40,41].

### Limitations

There are some limitations of this study that need to be acknowledged. Blinding of subjects could not be implemented, as each subject’s group allocation was obvious based on the subject’s physiotherapist conducting the training sessions through the respective intervention.

All subjects were recruited from the patient collective of a two-week inpatient multimodal pain therapy. In addition to the respective interventions, the test persons also received pain medicine treatments, exercise therapy and psychotherapeutic measures. Therefore, co-effects in the results cannot be excluded with certainty. Furthermore, it cannot be excluded that concomitant diseases may also have had an influence on the outcome of this study.

All subjects (n = 75) were randomly allocated to the three groups (n = 25 per group), each with different forms of sensorimotor training. Two groups (Galileo^®^ and Posturomed^®^) were defined as intervention groups. Since the control group also received standardized sensorimotor training under physiotherapeutic guidance, no group was disadvantaged with lesser therapeutic measures. However, this led to a lack of a true intervention-free control group. An intervention-free control group might have demonstrated clearer effects of sensorimotor training.

The inpatient setting in which the study was conducted limited the intervention period to two weeks. Furthermore, this study did not investigate whether the positive effects of the interventions are temporary or lasting. This would have required a follow-up study and potential for subject attrition.

Finally, lumbar stability was not assessed directly but via measurement of transversus abdominis activation in patients with chronic low back pain.

## 5. Conclusions

The results of this study demonstrate that the use of sensorimotor training in different forms can be effective in the rehabilitation of patients with chronic low back pain. More specifically, sensorimotor training on the Galileo^®^ may lead to greater improvement in activation of the transversus abdominis compared to sensorimotor training on the Posturomed^®^ or sensorimotor training through conventional physiotherapy (control group). This could justify a permanent establishment of sensorimotor training on the Galileo^®^ when using multimodal pain therapy. However, the use of supportive training devices should not replace the presence of medical professionals. As described in the present study, detailed instruction of the exercises by an experienced physiotherapist was necessary. The validity of using functional clinical diagnostics to determine the effectiveness of sensorimotor training on segmental stability could not be refuted in the present study. However, clinical diagnostics are and remain an important tool for clinicians. Future research should investigate how functional clinical diagnostics could be supplemented by more objective, sensory measurement methods of movement control in the evaluation of therapeutic measures for chronic low back pain patients.

## Figures and Tables

**Table 1:** Clinical tests with execution and evaluation criteria.

Clinical Test	Implementation	Evaluated as Conspicuous at
**Inspiration in** **supine position**	Spontaneous breathing	Thoracic high breathing
**Hip flexion in** **supine position**	Lifting both legs	Lumbar lordosis, cranialization upper body
**Trunk flexion in** **supine position**	Supine position with feet apart, upper body erect	Heels leave base, scapula does not lift off
**Hip abduction in** **lateral position**	Lift contralateral outstretch leg	Leg rotation
**Vele test in** **standing position**	Shift weight forward	No gripping function of the toes
**Matthias test in** **standing position**	Arm elevation to 90° with internal roation	Scapula winging, Retroflexion upper body
**Single leg stand**	Flex contralateral hip and knee	Pelvic tilt to the healthy side (Trendelenburg sign)

**Table 2:** Description of the investigated sample regarding age, sex and anthropometric parameters. Results reported as mean ± standard deviation (95% CI).

	Galileo^®^	Posturomed^®^	Control	Variance Analysis	
				*p*	η_p_^2^
**Age [years]**	58.3 ± 11.6 (53.8–62.9)	62.0 ± 11.8 (57.4–66.5)	59.9 ± 10.7 (55.3–64.4)	0.529	0.018
**Height [m]**	1.70 ± 0.10 (1.66–1.74)	1.72 ± 0.12 (1.68–1.76)	1.68 ± 0.07 (1.64–1.72)	0.343	0.029
**Weight [kg]**	79.6 ± 19.2 (72.9–86.4)	87.2 ± 15.9 (80.4–93.9)	81.7 ± 15.6 (75.0–88.5)	0.277	0.035
**BMI [kg/m²]**	27.6 ± 5.42 (25.5–29.6)	29.5 ± 4.76 (27.5–31.6)	29.1 ± 5.34 (27.0–31.1)	0.385	0.026
**Sex,** **male:female**	10:15	12:13	5:20	*p* = 0.105 Chi-Squared: 4.51	

Analysis of the functional status measured with the Oswestry Disability Index (ODI) was described elsewhere. The mean values between groups varied between 17.3 and 22.3 [14].

**Table 3:** Descriptive comparison (mean ± standard deviation), variance analysis and calculation of the effect size η_p_^2^ between examination 1 and 2 depending on groups. Relevance level: *p* < 0.05 and η_p_^2^ ≥ 0.10. Relevant differences are marked in bold.

Parameter	Patients with Chronic Low Back Pain (n = 75)						Variance Analysis					
							Group		Time		Group × Time	
	Examination 1			Examination 2			p	η_p_^2^	p	η_p_^2^	p	η_p_^2^
	Galileo^®^	Posturomed^®^	Control	Galileo^®^	Posturomed^®^	Control						
**Muscle Relaxed [cm]**	0.33 ± 0.12	0.30 ± 0.08	0.36 ± 0.09	0.32 ± 0.10	0.37 ± 0.11	0.37 ± 0.09	0.269	0.036	0.059	0.049	**0.019**	**0.105**
**Muscle Contracted [cm]**	0.49 ± 0.16	0.40 ± 0.13	0.50 ± 0.13	0.56 ± 0.17	0.55 ± 0.15	0.53 ± 0.10	0.288	0.034	**<0.001**	**0.225**	0.028	0.095
**Muscle Activation [%]**	0.52 ± 0.27	0.32 ± 0.22	0.43 ± 0.25	0.81 ± 0.48	0.53 ± 0.25	0.49 ± 0.29	**0.003**	**0.147**	**<0.001**	**0.265**	0.049	0.080

**Table 4:** Descriptive comparison (mean ± standard deviation) and effect size calculation (d) for clinical tests. Examination: exam. Relevant differences (d > 0.5) marked in bold.

Clinical Test	Galileo^®^			Posturomed^®^			Control		
	Exam 1	Exam 2	d	Exam 1	Exam 2	d	Exam 1	Exam 2	d
**Inspiration in supine position**	0.12 ± 0.33	0.00 ± 0.00	**0.73**	0.24 ± 0.44	0.08 ± 0.28	0.34	0.12 ± 0.33	0.06 ± 0.22	0.22
**Hip flexion in supine position**	0.04 ± 0.20	0.00 ± 0.00	0.40	0.68 ± 0.48	0.12 ± 0.33	**1.24**	0.18 ± 0.38	0.00 ± 0.00	**1.00**
**Trunk flexion in supine position**	0.24 ± 0.43	0.04 ± 0.02	**0.63**	0.60 ± 0.50	0.24 ± 0.44	**0.77**	0.50 ± 0.50	0.48 ± 0.51	0.04
**Hip abduction in lateral position**	0.16 ± 0.37	0.04 ± 0.02	0.42	0.20 ± 0.41	0.12 ± 0.33	0.22	0.16 ± 0.35	0.08 ± 0.28	0.25
**Vele test in standing position**	0.08 ± 0.28	0.00 ± 0.00	**0.57**	0.00 ± 0.00	0.00 ± 0.00	0.00	0.12 ± 0.33	0.04 ± 0.02	0.30
**Matthias test in standing position**	0.12 ± 0.03	0.00 ± 0.00	**0.73**	0.08 ± 0.28	0.04 ± 0.20	0.16	0.12 ± 0.03	0.04 ± 0.20	0.30
**Single leg stand**	0.00 ± 0.00	0.00 ± 0.00	0.00	0.08 ± 0.28	0.00 ± 0.00	**0.57**	0.04 ± 0.20	0.00 ± 0.00	0.40

**Table 5:** Pearson correlation coefficients (r) between clinical tests and the parameter “sonography M. transversus abdominis” (n = 75). Relevant correlations (r > 0.5) marked in bold.

Clinical Test	Activation M. Transversus Abdominis
**Inspiration in supine position**	−0.276
**Matthias test in standing position**	0.212
**Hip flexion in supine position**	−0.211
**Trunk flexion in supine position**	−0.178
**Single leg stand**	0.155
**Hip abduction in lateral position**	0.095
**Vele test in standing position**	−0.060

## Data Availability

Not applicable.
